# Supplementary material for: Unraveling the In Vitro Toxicity Profile of Psychedelic 2C Phenethylamines and Their N-Benzylphenethylamine (NBOMe) Analogues
Source: Pharmaceuticals (Basel). 2023 Aug 15;16(8):1158. doi: 10.3390/ph16081158 (PMC10458253; doi:10.3390/ph16081158)
Supplement: Supplementary file 1 [file pharmaceuticals-16-01158-s001.zip › pharmaceuticals-2526542-supplementary.pdf]

Supporting information

Unraveling the In Vitro Toxicity Profile of Psychedelic 2C Phenethylamines and Their *N*-Benzylphenethylamine (NBOMe) Analogues

Daniel Martins [1,†], Eva Gil-Martins [1,2,3,†], Fernando Cagide [1], Catarina da Fonseca [2,3], Sofia Benfeito [1],
Carlos Fernandes [1], Daniel Chavarria [1], Fernando Remião [2,3], Renata Silva [2,3,*] and Fernanda Borges [1,*]

[1] CIQUP-IMS/Department of Chemistry and Biochemistry, Faculty of Sciences, University of Porto, Rua do Campo Alegre s/n, 4169-007 Porto, Portugal; ester.benfeito@fc.up.pt (S.B.); carlos.fernandes@fc.up.pt (C.F.); daniel.chavarria@fc.up.pt (D.C.)
[2] Associate Laboratory i4HB—Institute for Health and Bioeconomy, Faculty of Pharmacy, University of Porto, 4050-313 Porto, Portugal; up201804901@edu.ff.up.pt (C.d.F.); remiao@ff.up.pt (F.R.)
[3] UCIBIO—Applied Molecular Biosciences Unit, Laboratory of Toxicology, Department of Biological Sciences, Faculty of Pharmacy, University of Porto, 4050-313 Porto, Portugal
* Correspondence: rsilva@ff.up.pt (R.S.); fborges@fc.up.pt (F.B.)
† These authors contributed equally to this work.

Figures

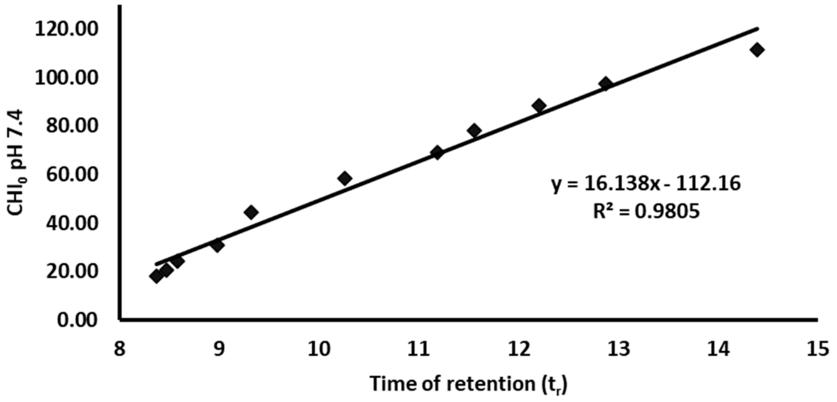

Figure S1. Calibration line obtained after plotting the individual retention times of the standard compounds as a function of their respective literature CHI values at pH 7.4.

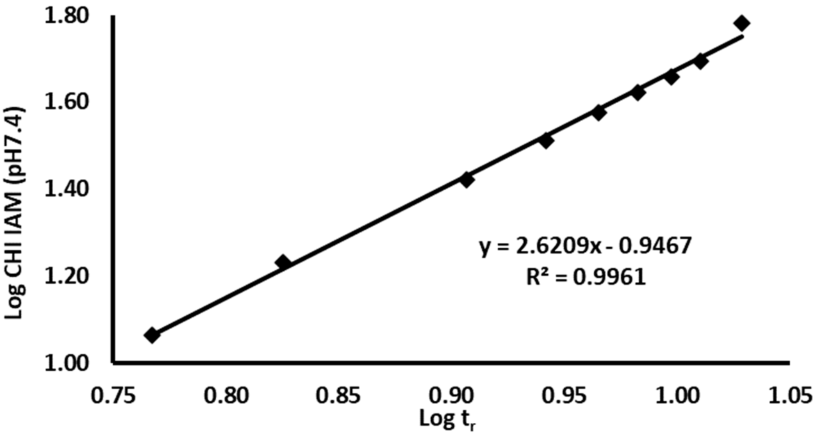

Figure S2. Calibration line obtained after plotting the logarithmic values of the individual retention times of the standard compounds as a function of the logarithmic values of their respective CHI (IAM) values at pH 7.4.

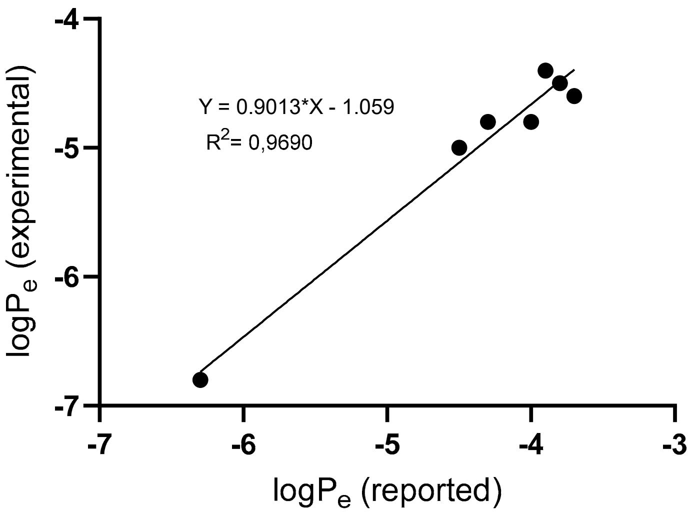

Figure S3. Calibration line obtained after plotting the controls experimental and reported PAMPA-BBB permeability (see data on the Table 2 of the manuscript).

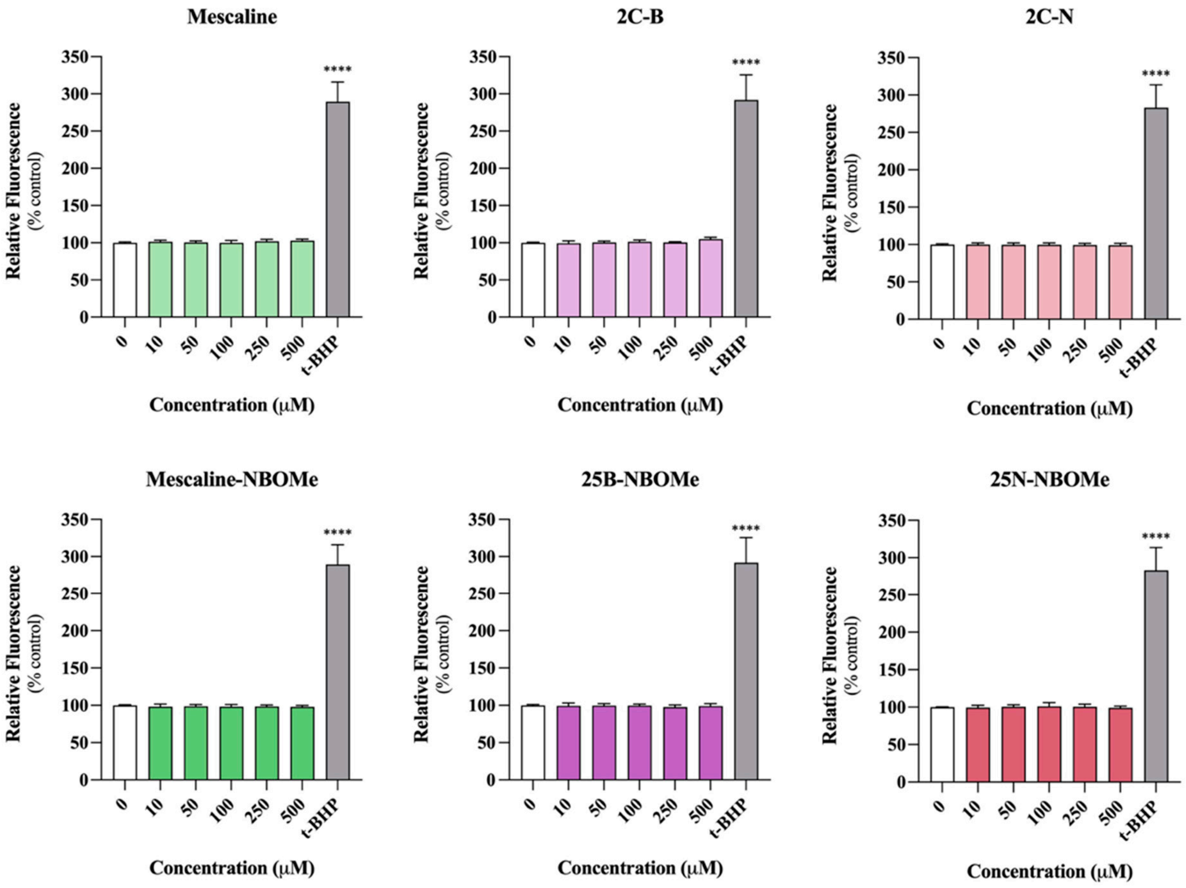

Figure S4. Intracellular reactive oxygen species (ROS) levels evaluated with DCFH-DA probe (10 μM), in differentiated SH-SY5Y cells, 24 hours after exposure to the tested drugs. Results are presented as Mean ± SD of at least 4 independent experiments (performed in triplicate). Tert-butyl hydroperoxide (t-BHP, 200 μM) was used as a positive control. Statistical comparisons were performed using One-way ANOVA, followed by Dunnett's multiple comparison post hoc test. [**** $p < 0.0001$ vs. control (0 μM)]. In all cases, p values < 0.05 were considered statistically significant.

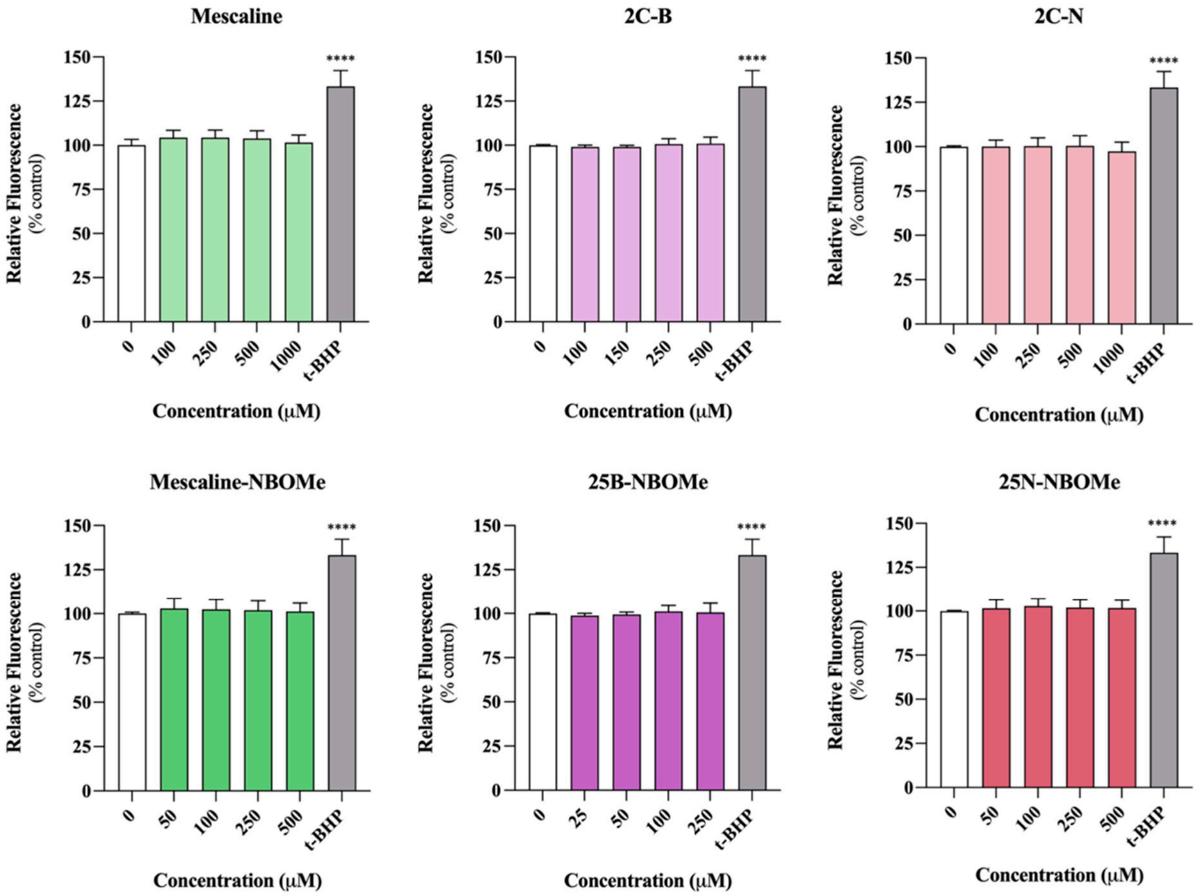

Figure S5. Intracellular reactive oxygen species (ROS) evaluated with DCFH-DA probe (10 μM), 24 hours after exposure of HepG2 cells to the tested drugs. Results are presented as Mean ± SD of at least 4 independent experiments (performed in triplicate). Tert-butyl hydroperoxide (t-BHP, 200 μM) was used as a positive control. Statistical comparisons were performed using One-way ANOVA, followed by Dunnett's multiple comparison post hoc test [**** $p < 0.0001$ vs. control (0 μM)]. In all cases, p values < 0.05 were considered statistically significant.

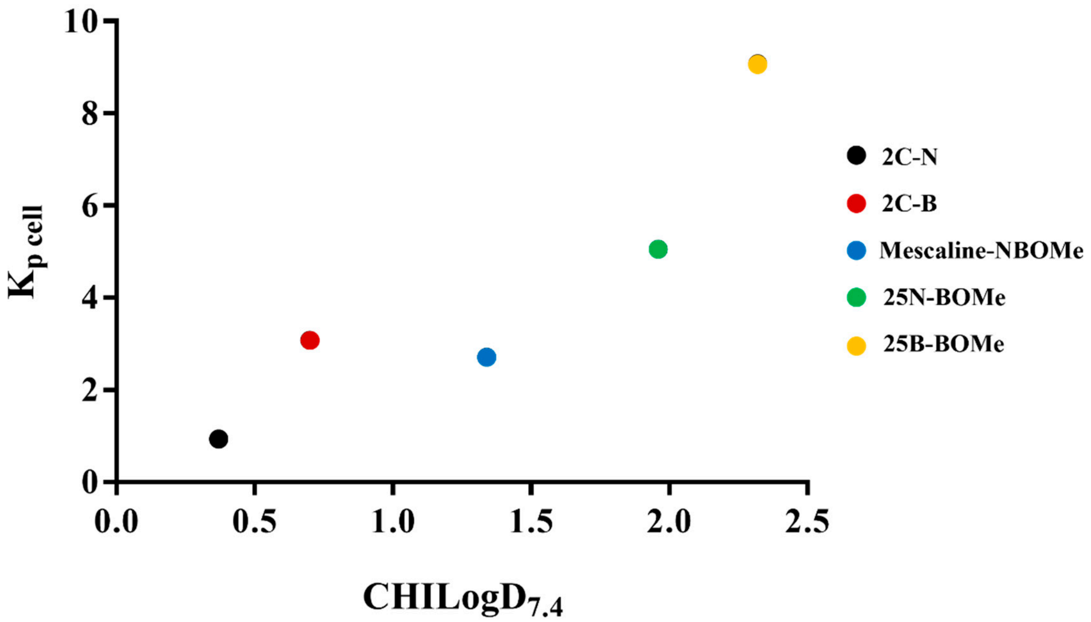

Figure S6. Correlation between the values of K_{pcell} and $CHILogD_{7.4}$ values.

Tables

Table S1. Individual literature CHI values of the of standards at pH 7.4 and their retention times (t_r) obtained by HPLC.

pH 7.4		
**Reference compound**	**CHI_0**	**t_r**
**Theophylline**	18.19	8.38
**Paracetamol**	20.59	8.47
**Caffeine**	24.12	8.58
**Benzimidazole**	30.71	8.98
**Colchicine**	44.32	9.32
**Carbamazepine**	58.45	10.26
**Indole**	69.15	11.19
**Propiophenone**	78.41	11.56
**Butyrophenone**	88.49	12.21
**Valerophenone**	97.67	12.88
**Heptanophenone**	111.8	14.40

Table S2. Individual literature CHI (IAM) values of the standards at pH 7.4, their retention times (t_{rIAM}) experimentally determined by HPLC and their respective logarithmic values.

	pH 7.4			
	t_r (min)	Log (t_r)	CHI (IAM)	Log [CHI (IAM)]
**Acetanilide**	5.85	0.77	11.57	1.06
**Acetophenone**	6.69	0.83	17.07	1.23
**Propiophenone**	8.07	0.91	26.39	1.42
**Butyrophenone**	8.746	0.94	32.55	1.51
**Valerophenone**	9.226	0.97	37.71	1.58
**Hexanophenone**	9.611	0.98	41.91	1.62
**Heptanophenone**	9.939	1.00	45.49	1.66

Table S3. Evaluation of *h*MAO inhibitory activities of the drugs under study and reference MAO inhibitors.

	% inhibition at 10 µM or IC_{50} (µM)		SI[a]
**Compound**	***h*MAO-A**	***h*MAO-B**	
**Mescaline**	2.5 %	21.2 %	—
**Mescaline-NBOME**	3.9 %	89.0 %	—
**2CB**	17.5 %	27.2 %	—
**25B-NBOME**	15.0 %	33.9 %	—
**2C-N**	5.8 %	18.0 %	—
**25N-NBOME**	8.9 %	25.5 %	—
**Clorgyline**	0.00260±0.00033	1.93±0.16	0.00135
**Rasagiline**	3.72 ± 0.38	0.149 ± 0.023	24.9

[a] SI: *h*MAO-B selectivity index = IC_{50}(*h*MAO-A)/IC_{50}(*h*MAO-B).
